# **Initial findings creating a temperature prediction model using vibroacoustic signals originating from tissue needle interactions**

**DOI:** 10.1038/s41598-025-92202-6

**Published:** 2025-03-03

**Authors:** Michael Friebe, Witold Serwatka, Katharina Steeg, Gabriele Krombach, Hamza Oran, Oğuzhan Berke Özdil, Katarzyna Heryan, Axel Boese, Alfredo Illanes, Dominik Rzepka

**Affiliations:** 1 Faculty of Computer Science, AGH University of Kraków, al. Adama Mickiewicza 30, 30-059 Kraków, Poland; 2 University of Giessen, Ludwigstr. 23, 35390 Giessen, Germany; 3 Universitätsklinikum Giessen and Marburg, Klinikstr. 33, 35392 Giessen, Germany; 4 INKA Innovation Lab, Faculty of Medicine, Otto-von-Guericke-University, Leipziger Str. 44, 39120 Magdeburg, Germany

**Keywords:** Tissue temperature, Thermotherapy, Thermal monitoring, Minimal-invasive therapy, Vibroacoustic signals, Diagnostic markers, Biomedical engineering, Acoustics, Characterization and analytical techniques

## Abstract

This research explores the acquisition and analysis of vibroacoustic signals generated during tissue-tool interactions, using a conventional aspiration needle enhanced with a proximally mounted MEMS audio sensor, to extract temperature information. Minimally invasive temperature monitoring is critical in thermotherapy applications, but current methods often rely on additional sensors or simulations of typical tissue behavior. In this study, a commercially available needle was inserted into water-saturated foams with temperatures ranging from 25 to 55 °C, varied in 5° increments. Given that temperature affects the speed of sound, water’s heat capacity, and the mechanical properties of most tissues, it was hypothesized that the vibroacoustic signals recorded during needle insertion would carry temperature-dependent information. The acquired signals were segmented, processed, and analyzed using signal processing techniques and a deep learning algorithm. Results demonstrated that the audio signals contained distinct temperature-dependent features, enabling temperature prediction with a root mean squared error of approximately 3 °C. We present these initial laboratory findings, highlighting significant potential for refinement. This novel approach could pave the way for a real-time, minimally invasive method for thermal monitoring in medical applications.

## Introduction

### Thermotherapy background

Human blood and cells are very temperature sensitive. Lower as well as higher than 37 °C can have positive and negative effects. Several interventional, minimal-invasive therapies take advantage of that for manipulating perfusion, cell killing and tissue destruction or removal^1^.

Hypothermia (temperatures of around 33 °C can inhibit coagulation and improve microcirculation as well as reduce formation of micro thrombi in the brain^2^. Cell killing hyperthermia therapies apply temperatures between 42 and 48 °C that cause irreversible cell damage proportional to the total time applied^3^.

Ablation therapies typically use temperatures between 65 and 75 °C for durations of 5–15 min^4^. Cryotherapies on the other hand require a freezing temperature (typically below − 50 °C or even lower temperatures for longer times) for cell killing^5^.

For all of these therapies it is necessary to obtain at least qualitative and preferably quantitative temperature measurements. This is typically achieved through interstitial temperature sensors or through external temperature control with imaging systems (e.g. temperature sensitive MRI sequences)^6,7^.

The placement of additional needles is invasive and while the temperature monitoring with MRI is feasible these clinical procedures are rarely done in a radiological suite (MRI or CT) that is not prepared for therapeutic applications. For use with a MRI system all interventional components would need to be MRI compatible (non-ferrous material, e.g. certain Co–Cr alloys or better Nitinol) and all electronics would need to be able to work in very strong magnetic fields.

In many cases the temperature variation is merely simulated based on lab results that are not properly considering individual tissue behavior, as well as tissue perfusion and diffusion^8^.

In-vivo tissues are more or less heavily perfused, which leads to internal tissue cooling and makes temperature simulations so difficult and at the same time the requirements for local temperature measurement so important.

A method that would be able to do a real-time qualitative measurement of temperature changes without a dedicated thermometry sensor or external imaging would present a less invasive approach and one that actually measures rather than simulates a temperature distribution.

Adding temperature sensors to the tip of the interventional device is a possibility, but this requires a dedicated and likely expensive system with electric cables inside the patient. And it likely also diminishes the actual operational functionality of the device as it takes up space at the tip.

An ideal scenario would be to use existing and proven (certified) devices that can be retrofitted with an additional sensor unit that does not reduce functionality of the core device. A normal temperature unit would not be suitable as it would not be able to measure at the most important location of the device, at the tip.

### Tissue heating: molecular effects

When water molecules in biological tissues are heated, several physical and structural changes occur. Initially, heating causes an increase in tissue temperature, leading to changes in the mechanical properties of the tissue. As a result, the tissue’s extensibility, hysteresis, and compliance increase, indicating that the tissue becomes more flexible and less stiff for temperatures below 43 °C^9^.

Collagen, a major component of connective tissues, undergoes denaturation when heated over 61 °C. This process transforms its native helical structure into a more random, coiled structure, increasing the entropy of the protein^10^.

Additionally, heating can lead to the evaporation of water within the tissue, causing desiccation and further altering tissue properties^11^. This water loss can affect the tissue’s electrical conductivity and specific heat, which are temperature-dependent^12^.

At higher temperatures (over 60 °C), such as those used in certain medical therapies, the structural integrity of in-vivo tissues can be compromised due to collagen denaturation and tissue shrinkage^13^. These changes can influence the clinical outcomes of treatments like laser or microwave therapies, as the post-treatment mechanical properties of the tissue are crucial for recovery and repair.

### Sound, acoustics and temperature

Conventional medical ultrasound, using speed of sound, has been used for thermal monitoring using a speed-of-sound tomographic approach coupled with a biophysical heat diffusion model. The relation between changing temperatures, changing tissue behaviors exposed to temperature changes^14,15^ and the speed of sound for ultrasonography are established and observable^16,17^. Using acoustic signals for temperature measurement and thermography monitoring comes with many drawbacks though, mainly complexity^18^, loss of pixel information, relatively inaccurate temperature results, they are limited to temperatures before tissue coagulation (60 °C), they require expensive devices and dedicated evaluation equipment.

Some of these issues can be resolved by applying machine learning approaches^19^, but there are still many open research questions that have prevented that approach from being used in-vivo up to now and it is still necessary to transmit reference signals.

Acoustics are also used in vibroacoustography, a speckle-free ultrasound based imaging modality that can visualize normal and abnormal soft tissue through mapping the acoustic response of the object to a harmonic radiation force induced by ultrasound that might be usable for thermal measurements^20^.

Our own past research dealt with acquisition and evaluation of vibroacoustics obtained through tissue—device interactions (e.g. moving an aspiration needle in selected tissues), using a proximally placed microphone attached to the needle shaft^21^.

**Fig. 1:**
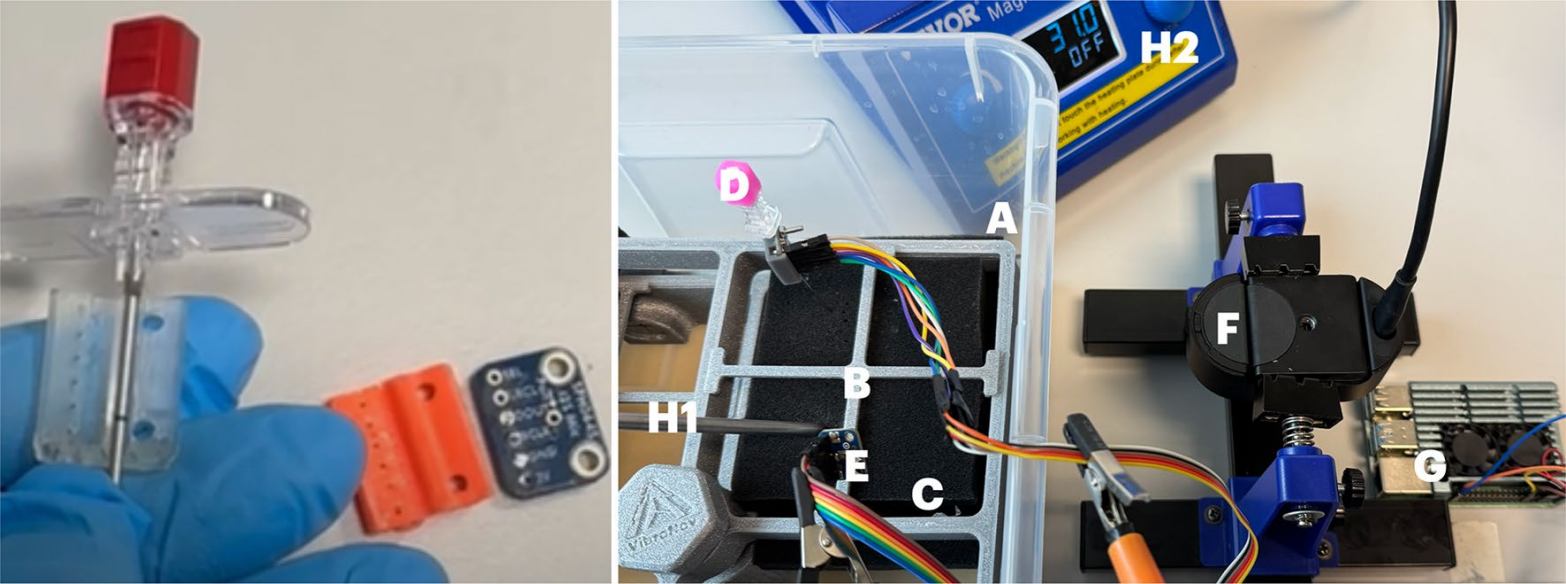
Left: A conventional and commercially available aspiration needle (Quincke 22G 9 cm Length) was retrofitted with a proximally placed (outside a “patient“) MEMS microphone (SPH0645) using a 3D printed adaptor. Right: The experimental setup with a waterbath (**A**) with a holding frame (**B**) for the foam (**C**), the needle with the attached microphone (**D**), an external microphone (identical to the one on the needle) to record any ambient noise (**E**), videocamera to record the insertions (**F**), the Raspberry Pi unit (**G**), and (**H1**) the temperature sensor (PT100) and the temperature measurement unit (**H2**). The foam (**C**) was water saturated (32% by volume) and the waterbath was slowly allowed to cool down from 55° while the needle insertions and the recordings were taken. The vibroacoustic recordings were placed in 5° groups.

For that we developed a dedicated 3D printed attachment for the audio sensor and recorded the data for event and tissue characterization, generated during the insertion of the needle in different artificial and animal tissues, with the goal to enhance and support device navigation in tissues (see Fig. 1 left). The audio data was filtered and preprocessed via a directly attached raspberry pi unit before sent to a processing unit.

In that research we were able to detect certain events (e.g. punctures, different tissues) and also classify some tissues based on the audio signals received on the proximal end of the needle. This placement may diminish the signal quality, but it also allows feasibility using standard, certified and approved, devices/tools, is relatively cheap and easy to attach, and also does not require any cables that are inserted into the patient^22,23^.

The research also showed that vibroacoustic signals contain information about tissue interactions that will change with the mechanical properties of the interacted tissue. A more dense or a softer tissue will create a different acoustic response when interacting with a clinical device.

We know that the tissue properties change with temperature increases and decreases. They might therefore also affect the vibroacoustic signal and with provide an indirect way to obtaining temperature changes.

This led us to the formulation of our research hypothesis based on the clinical need to provide least invasive thermal monitoring as part of a thermotherapy.

### Research hypothesis

Our past research showed that we were able to use vibroacoustic signals for device guiding and event characterisation using a MEMS microphone sensor attached to the proximal end of a clinical interventional device. We specifically used that location as a compromise between ease of implementation and subsequent clinical application (no dedicated system, just a clip-on to existing devices), signal quality (sensor placement at the tip is likely better), and regulatory complexity (sensor placed outside the body, without active components or wires inside the body). As many of the involved physical principles and tissues are temperature dependent we believe that the obtained acoustic signal could carry a temperature derivative that would allow identification of temperature changes and possibly even quantitative temperature information in the range relevant for Hypothermia and Hyperthermia applications.

This lead to the research hypothesis^21–23^:

*Proximally acquired vibroacoustic signals, obtained as part of the tissue interaction with an interventional device, contain temperature dependent information that can be extracted and visualised using signal processing and deep learning models in a relevant temperature range from around 30 °C to 50 °C.*

## Materials and methods

The problem is that ex-vivo tissues do alter significantly over a very small temperature range without perfusion and without the controlling functions of a live body.

This makes an initial experimental setup using ex-vivo animal tissues very challenging, if not impossible. Identifying and using materials that are stable in their behaviour and mechanical properties over the temperature range of interest, while containing as much water as possible in a stable structure is essential. This is of course necessary, as we want to ensure that any observed signal deviations have their origin in or are directly related to the temperature change.

**Fig. 2:**
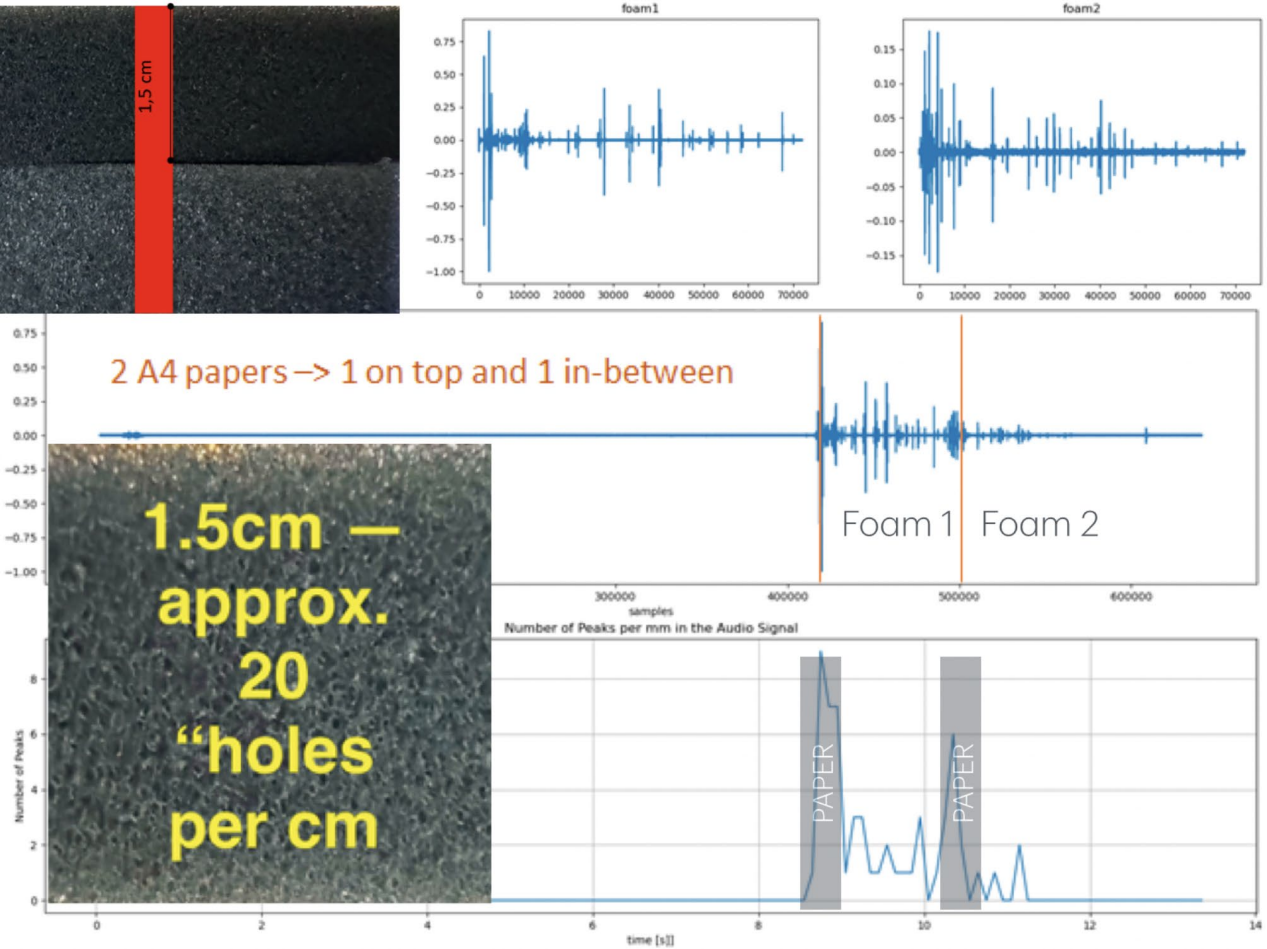
The reference phantom that we used to verify the general functionality consists of two different foams (Black Cut PUR RG25 and RG40). If you observe the foam structure you can identify around 20 bubbles per cm in foam 1 and around 8–10 bubbles in foam 2. Every time the needle crosses/penetrates such a bubble an acoustic event is created that can be detected as a signal peak. The picture above left shows the two different foams, each 1.5 cm, and the needle path (red). The corresponding acoustic signals are shown on the top right. The initial strong signals originate from the penetration of a normal piece of paper placed on top of the foams. On the bottom left you see a scale up of one of the foams. We developed a peak counting algorithm (peaks over time—see bottom right) for this setup to ensure a repeatable signal acquisition quality (signal amplitude and number of peaks). As indicated we were able to count ~ 20 peaks/cm for Foam1 and ~ 9 peaks/cm for foam2.

We identified a commercially available foam (Black Cut PUR RG25) as an ideal reference and artificial test tissue. Depending on the type of foam it contains a relatively accurate number of air bubbles per cm. If these air bubbles are crossed or penetrated by the needle passing through, a vibroacoustic signal is generated that can be detected as a peak in the audio data via a microphone attached to the proximal end of the needle. We developed a peak counter algorithm identifying the number of peaks per time that corresponds very closely with the actual air bubbles per cm. The foams, audio waveforms for the different foams, a close-up look at one of the foams (the one shown has about 20 holes per cm) and the peak counter are shown in Fig. 2.

Our experiments were conducted in a temperature range that will likely not affect the chosen foams mechanical behavior being well below the boiling point of water.

This general setup allowed us, in a consistent and repeatable setup, to calculate the events per distance that are directly correlated to the punctured material. Variation in insertion speed did not affect the peak count per cm. These foams were also used for the temperature measurement experiments and showed to behave exactly the same behavior water saturated with respect to the number of peaks per cm.

The experimental setup chosen for the manual insertion is shown in Fig. 1 right with a waterbath (A) with a holding frame (B) for the foam (C), the needle (Quincke 22G 9 cm Length) with the attached microphone (D) (SPH0645 MEMS), an external microphone (identical to the one on the needle) to record any ambient noise (E), videocamera to record the insertions (F), the Raspberry Pi unit (G), and (H1) the temperature sensor (RTD point sensor PT100 with an accuracy of better than 0.1 °C in the temperature range from 10 to 100 °C) and the temperature measurement unit (H2). The foam (C) was water saturated (32% by volume) and the waterbath was slowly allowed to cool down from 55° while the needle insertions and the recordings were taken.

**Fig. 3:**
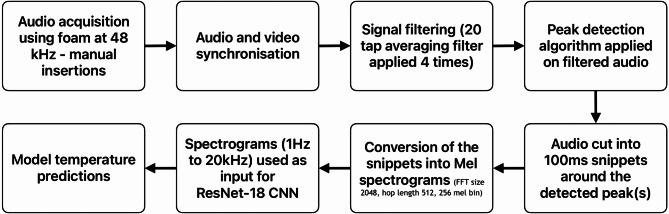
The experimental workflow and the individual processing steps from the needle insertion (top left) to the temperature prediction.

The process from the needle insertion to the temperature prediction is shown in Fig. 3 and the individual steps are subsequently described.

**Fig. 4:**
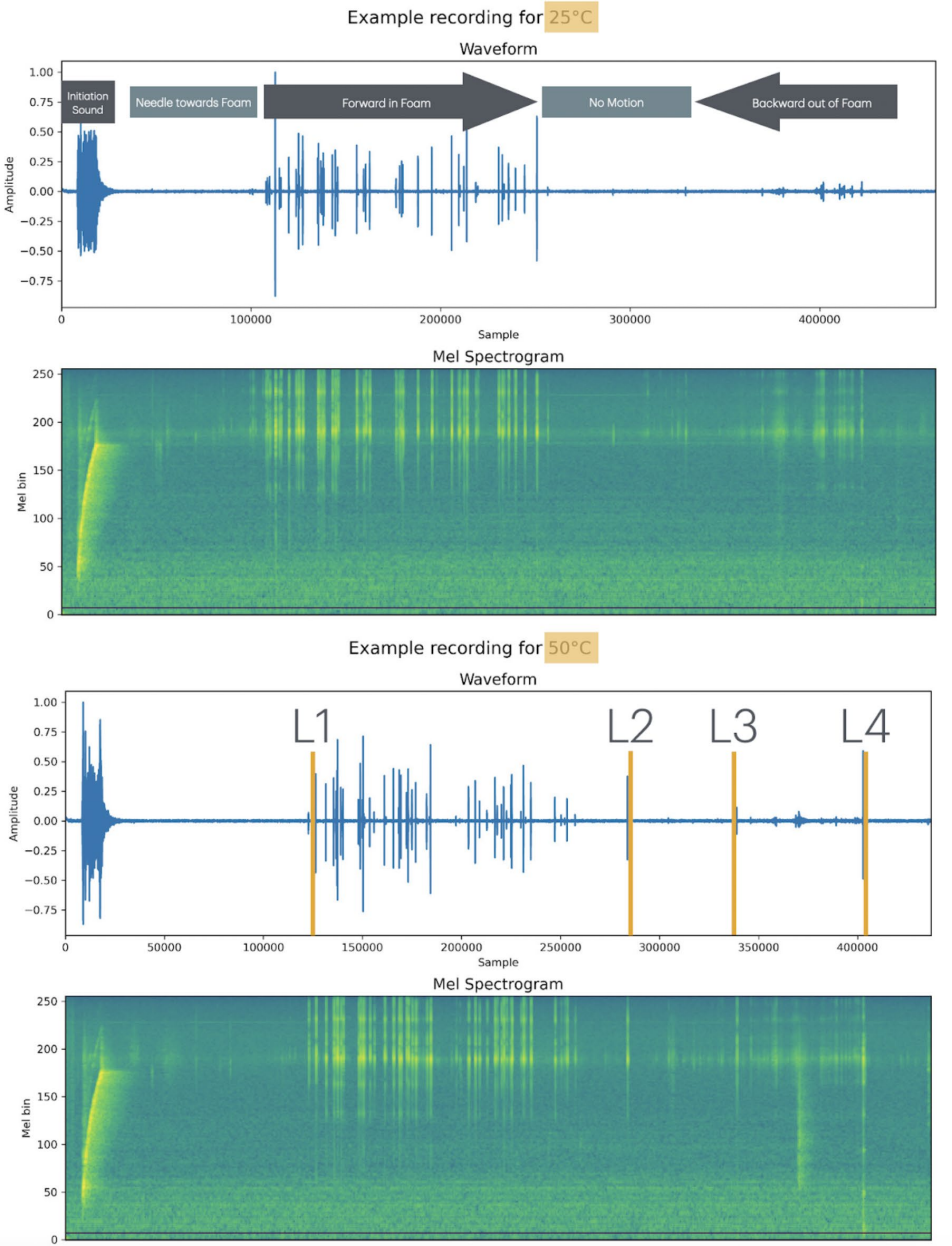
Top: 25° audio recording with the raw amplitude over time (48 kHz sample rate) and the respective mel spectrogram below it. Every insertion starts with an initiation sound as shown on the left, followed by a certain period of time it takes to move the needle towards the foam, the forward motion inside the foam, a waiting time after forward motion is ended and the backward motion. Bottom: Raw signal and mel spectrogram for the 50° recording and the annotations used for the deep learning : L1 = label for entering the foam (verified by the video camera), L2 indicates the stop of the forward motion, L3 the begin of the needle retrieval and L4 the point in time when the needle exits the foam again.

The needle equipped with the proximally attached microphone is inserted in the water saturated foam. An example of the obtained raw audio signal profiles and the respective mel spectrogram (acquired at 48 kHz sample rate) for two different temperatures with the Labels L1–L4 for a temperature of 25 and 50 °C is shown in Fig. 4.

A video camera was installed for subsequent annotation support and the event labeling of the time of foam puncture (L1), end of the forward puncture motion (L2), start of needle removal (L3), and exit of needle from the foam (L4) as shown in Fig. 4.

We used a manual insertion speed of around 5–10 mm/s and started every sequence with an initiation audio pulse (seen on the left of the raw data profile and the mel spectrogram in Fig. 4), the actual needle movement was between 7 and 12 s. The needle does not move between L2 and L3 with the relevant data that was used for the deep learning being between L1 and L2.

We acquired a total of 179 full recordings (around 22 each for each of the 5 °C windows from 20 to 55 °C). The audio signals were gathered at the beginning of each of these 5° intervals, while the actual temperature from beginning to end of each interval varied by up to 1.4 °C due to the ambient cooling effects (obviously more with higher temperatures and less for 30° and lower).

All audio recordings were synchronized with a video recorded during the puncture procedure. This was used to later annotate the beginning and end of the needle’s forward motion inside the foam.

Initially, these audio recordings, together with the annotations, were used to create a dataset of 100 ms long waveform snippets with a 25% overlap, converted into Mel spectrograms.

Having found the first results promising, we moved on to a peak-based method, both in order to minimize the possibility of fitting to non-temperature related features (e.g. room noise) and to counteract annotation errors.

The dataset used for discovering the findings of this paper was created by detecting the peaks in the audio waveform. The idea behind this was to ensure that the model focused on amplitude changes created by the needle’s passage through the foam.

To detect peaks we applied a 20 taps averaging filter 4 times to the rectified signal, to find the smoothened envelope. Then all local extrema were located. All local maxima in the audio signal were considered containing random noise and peaks resulting from the interaction of needle with foam (true peaks). The rationale behind this heuristics is that their amplitudes approximately follow normal distribution with the noisy peaks being the ones close to the mean, while true peaks must have a sufficiently large amplitude. We assumed three-sigma rule as a point of reference, and experimentally verified that setting threshold at one sigma above the mean gave acceptable result.

Using mean \mu and standard deviation \sigma of the peak amplitudes we obtained the experimentally validated threshold \theta = \mu + \sigma, to discard small peaks resulting from background noise.

Next, the recordings were sliced into 100 ms chunks. To augment the total amount of data the detected peaks were positioned approximately at the center of each 100 ms chunk, with a random (uniform) displacement of up to 25% of the slice width to the left or right, introducing an element of data augmentation. This displacement was implemented using the numpy.random.randint [https://numpy.org/citing-numpy/] function, which generates random integers uniformly within a specified range. Specifically, the displacement was sampled from the range [− 25%, + 25%] of the slice width, ensuring a uniform distribution of peak positions within this interval.

**Fig. 5:**
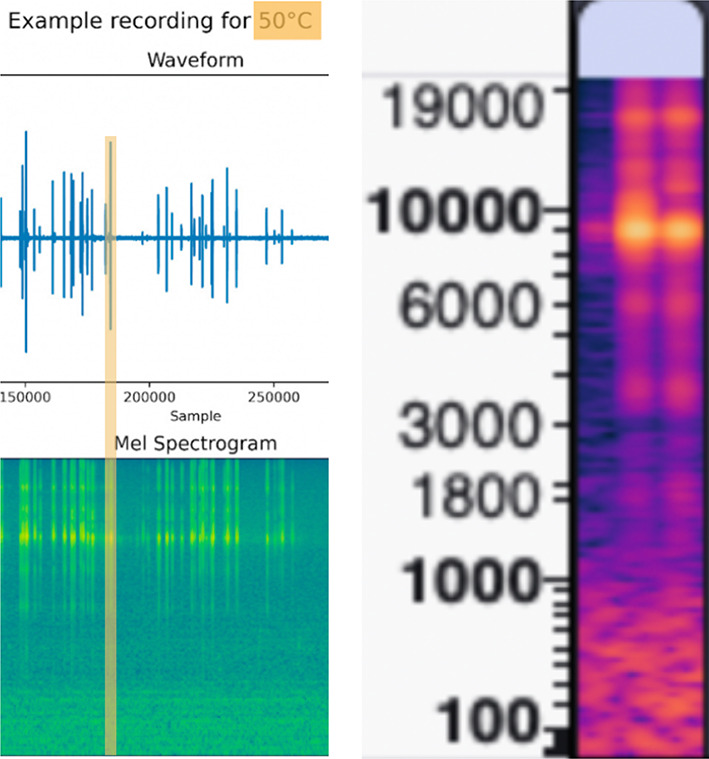
An example of a 100 ms slice extracted from the filtered audio data file using the peak detection (orange slice) subsequently converted into a mel-spectogram in frequency range from 1 Hz to 20 kHz. These slices were subsequently used for training the Resnet neural network.

In total, 4393 chunks were created and subsequently converted to Mel spectrograms (FFT size 2048, hop length 512 and 256 mel bins). Figure 5 shows an example a spectogram slices at one of the peak locations identified by the peak detection algorithm.

All the models were trained using fivefold cross-validation and the dataset was split into fivefolds of approximately equal size. While the split was selected randomly, it was ensured that spectrograms stemming from a common recording were part of the same fold in order to remove the risk of data leakage.

The training was done with data augmentation techniques, a gain variation from − 5 to 5 dB, pitch shift from − 3 to 3 dB, and time stretching in order to assure the model did not overfit on our dataset and was overall robust.

A ResNet convolutional neural network [https://arxiv.org/abs/1512.03385] in 18-layer version, pretrained on ImageNet dataset was used as a backbone. We used the smallest Resnet variation because of the small size of the dataset, as well as it’s proven effectiveness in similar tasks [Seibold, Matthias, et al. “Real-time acoustic sensing and artificial intelligence for error prevention in orthopedic surgery”. Scientific Reports 11.1 (2021): 3993. The model head was modified for a regression task by changing the final layer from the default 1000 outputs to just 1.

The models were trained for 70 epochs using 32 batch size, using the AdamW optimizer, and the Cosine Annealing Learning Rate Scheduler with an initial learning rate of 0.001 and a final learning rate of 0.0001.

**Fig. 6:**
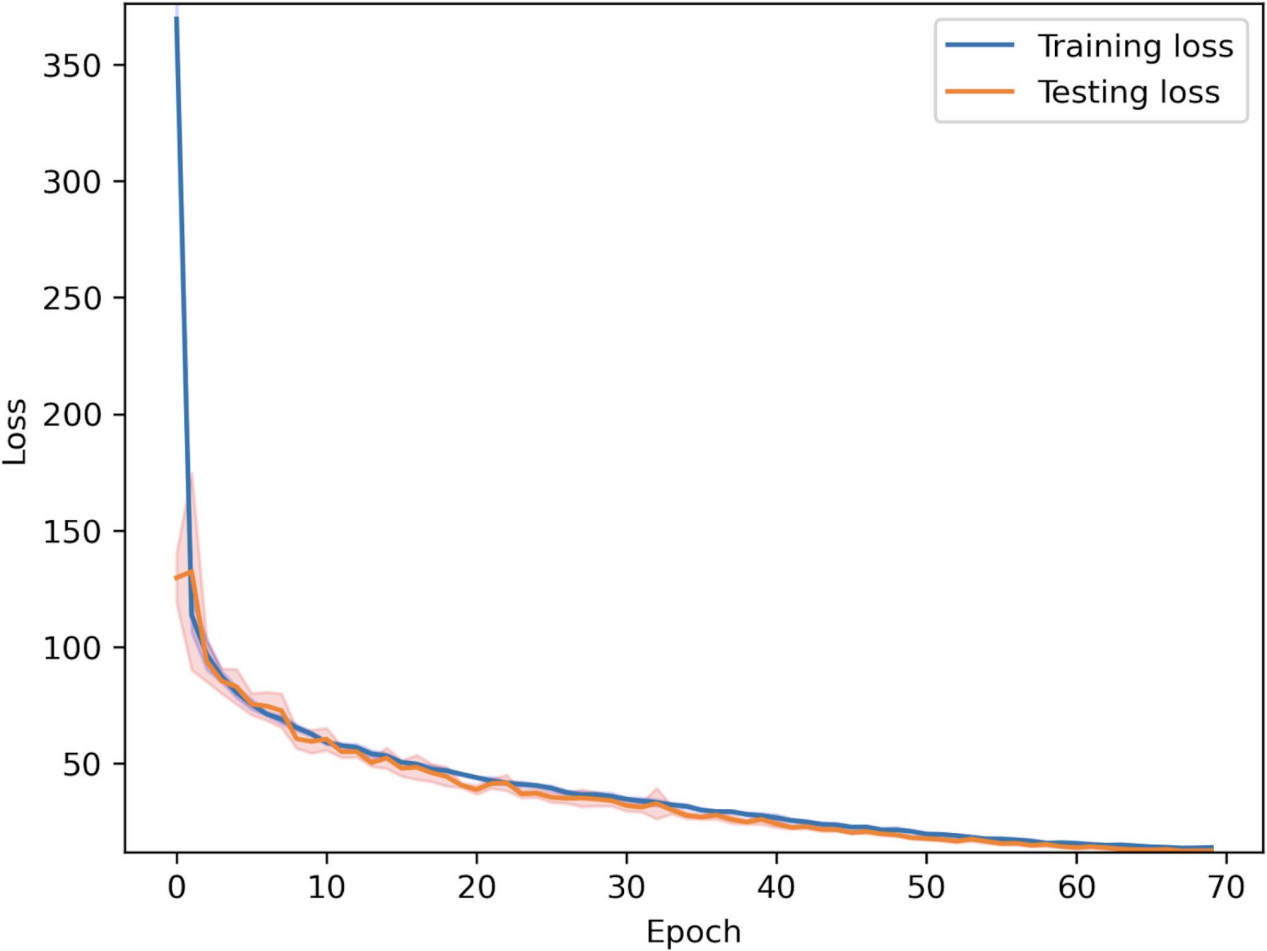
The train (blue) and test (orange) mean squared error (MSE) losses for the models trained on the peak-based dataset. The shaded areas show the 95% confidence interval for the values.

Mean squared error was selected for the loss function. Figure 6 depicts the changes in the training and test losses, implying no overfitting on the dataset.

## Results

The models trained on the peak-based dataset achieved a best RMSE value of 3.36 °C, with an average best RMSE of 3.4352 ± 0.0597 (± 1.74%). See Fig. 6 for the training/testing loss.

For the experiment we did not use a constant temperature, but started with the listed temperature of the individual ranges (e.g. with 45° for the 45° range) and subsequently measured during the cool-down of the waterbath stopping at 20–25 measurements per interval. The cooldown went faster for the higher than for the lower temperatures intervals and as such the maximum temperature variation in the 55 °C was ± 0.7 °C.

For clinical applications a temperature resolution with separate and dedicated temperature sensors of 1 °C would be sufficiently precise^24^, but it is difficult to achieve this even with added invasive sensors. This 1° measurement accuracy would be sufficient for all the previously described applications.

Considering the temperature variation during the experiments of up to 0.7 °C and the fact that we used standard, not specifically fine-tuned, tools and equipment we believe that the obtained results are a very promising and provide an exciting base for future developments to reach a goal of ± 1 °C in clinical use over the relevant temperature range.

**Fig. 7:**
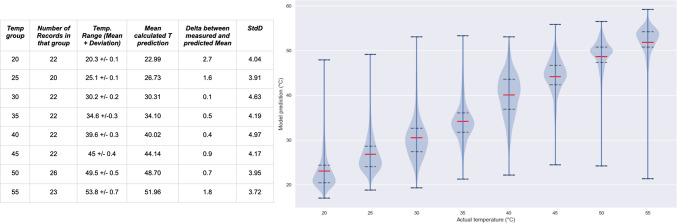
The table on the left shows the results for the individual temperature groups, including the mean of the measured temperature, the predicted mean including the delta between these two values, as well as the standard deviation for each of the temperature ranges. The violin plot shows the temperature predictions of the model plotted against the actual recorded temperatures. The middle (orange) line depict the mean of the predictions, while the dotted lines mark the 1st and 3rd quartiles, showing a clear correlation between the actual and predicted temperatures.

The table on the left of Fig. 7 presents the measured actual temperature range, the mean and deviation of this range, as well as the mean of the calculated prediction and the resulting delta between the measured and predicted temperature, which a maximum of 2.7 °C, minimum of 0.1 °C, and mean over all temperature ranges of 1.1 °C.

Figure 7 also shows a violin plot on the right, in which we can see the model’s temperature predictions plotted against the actual temperatures. The mean temperatures and standard deviations for each temperature range is listed in this figure as well.

**Fig. 8:**
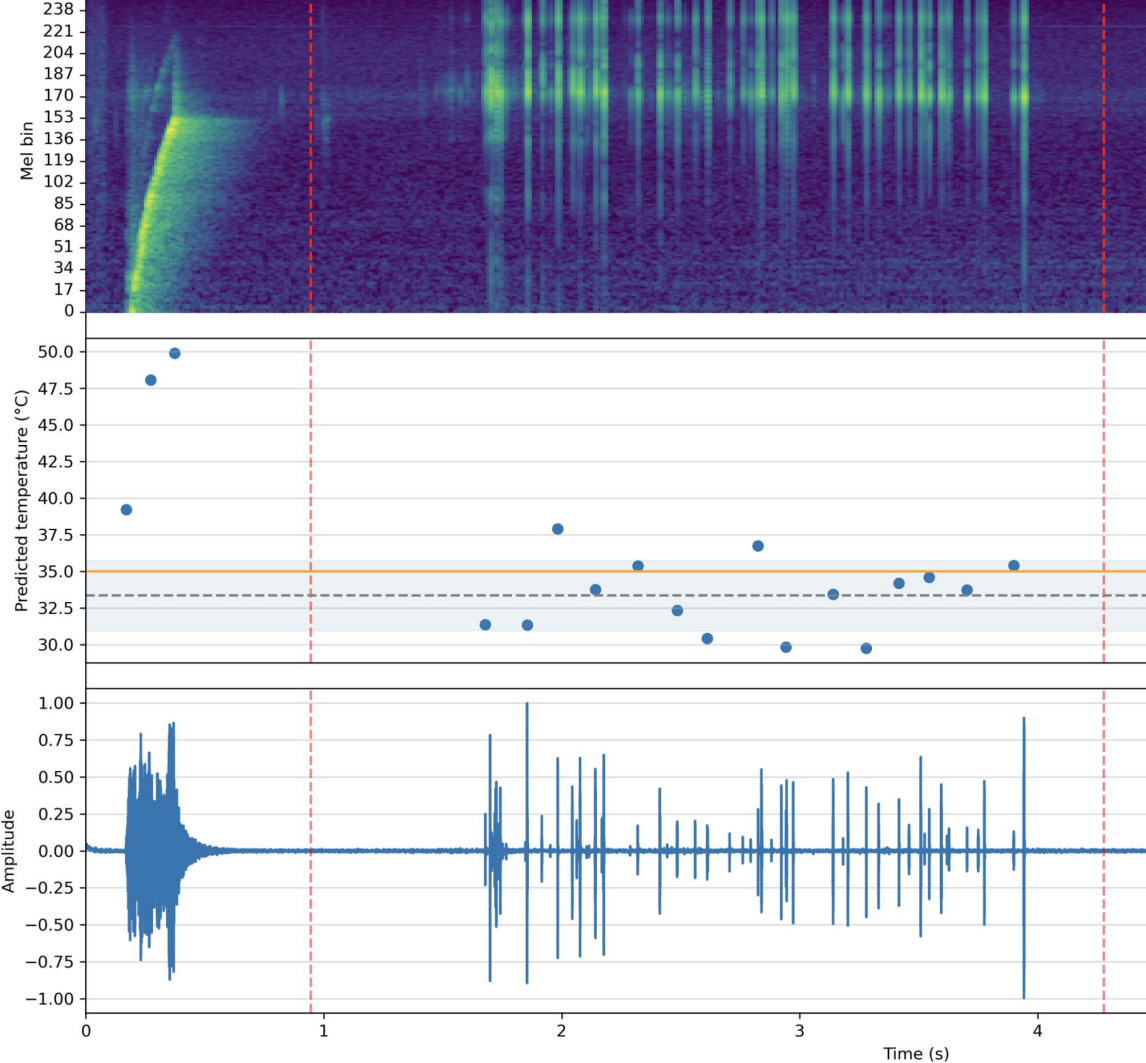
Example temperature prediction (here for a data set from the 35 °C group) in the middle. The points are identified for which the 100 ms mel spectrogram slices were identified (extracted from the top) based on the peak detection of the raw audio data set (bottom—between the two red dashed lines). The predicted temperatures for the initiation pulse (the three left ones) are not producing the correct prediction (the needle has not moved yet).

Figure 8 shows an example prediction for one of the 35° measurements with the temperature predictions using the mel spectrogram slice at the identified peaks from the raw audio data set.

## Discussion, conclusion and next steps

The advantage of the current setup that we propose is that certified and already available clinical interventional devices could be used with just a commercially available MEMS sensor microphone attached to the proximal end of the device (see Fig. 1) and some form of audio receiver hardware. While it might improve the obtained audio signal with the audio sensor placed at the tip of the interventional device it comes with many disadvantages (cables inside the patient, reduced functionality at the tip, sterility issues, dedicated device development needed including regulatory approval) that make this approach not feasible for the time.

We were able to show that vibroacoustic signals generated between the needle and the water saturated foam by manually moving (approximate forward/insertion speed of 5–10 mm/s) can be acquired with a standard microphone at the proximal end (outside the material or in a clinical process outside the patient). And, we also demonstrated in previous research that these signals can identify events like tissue puncturing and moving from one distinct material to another one.

The presented results in this research paper additionally showed that the same signal that is used for event and tissue characterization is able to identify temperature changes with a RMS error of 3.43 °C.

With larger number of parameters we are expecting problems with overfitting, as well as inference speed. To investigate this we are planning to investigate bigger networks in further research.

These results were obtained despite a rather simple tissue-simulating laboratory setup, without a properly controlled waterbath and with that the need to accept a wide temperature variation throughout the experiments.

We know for example from unpublished research that the cut and the surface of the needle tip has a large influence on the acoustic signal energy. And we also have confirmed that the signals generated at the tip are the ones that produce most of the relevant characteristic acoustic profile while the tissues that the needle shaft passes through are almost negligible. The needle insertion was done manually with varying insertion speeds, which is the likely setup and process in a clinical environment.

This of course leads to variations in the signal acquisition. We have also shown that this (in a normal bandwidth of user variation) does not affect the analysis though. Punctures such as the one in the foam phantom result in high frequency component dynamics. Any vascular pulsation picked up as audio in a future in-vivo use, even if it is big, will result in completely separable low frequency components^21,23^.

We have already started using a robotic system ensuring consistent insertion speeds and pressure. Such a setup would also enable us to implement an automatic labelling and segmentation approach. The main issue with that is the acoustic noise of the robot added to the signal.

The current approach of obtaining the audio signals is limited to the interventional device (needle) being in motion causing the needed friction for the audio signal creation. There might be technological solutions that could actively stimulate the tissues while the needle is not in motion to support the creation of an acoustic response of the tissue. We feel that this would make the entire process too complicated again and with that not realistic for a transfer into a real clinical environment.

The time and audio resolution show that forward (and backward) motions of around 0.5 mm are sufficient to obtain the needed information though.

The PU foams that we used show an effect of temperature on the mechanical properties only on a macroscopic level^25^. There is no research on the potential change of the microstructures and there is also no research, if the structures are filled with water and that water is heated or cooled.

While we do not believe that there are any sizable or relevant temperature related effects on the material in that small temperature range, we also cannot completely rule them out though.

The foam was fully saturated containing around 32% of water. While this is lower than in most human tissue, the temperature coefficient of tissue and the changes are higher than in the foam. With that the temperature derivative in the obtained vibroacoustics is very likely even more pronounced.

We also need to understand more about which part of the signal, or which measure, or which combination is responsible for the temperature characterization. Saliency maps and other model explainability techniques might lead to more insights.

We could then focus on these identified and more prominent parameters, fine-tune the signal processing or create different surface characteristics for the needle, optimize the microphone coupling or employ other strategies to enhance these most relevant features.

When dealing with ex-vivo (and subsequently with in-vivo) tissues in the future the effect of perfusion and the structural effects on viscoelasticity and hysteresis need to be carefully evaluated especially for temperatures over 60 °C.

The current approach is still very ineffective, time consuming, and far away from a real-time application. It is still unclear whether we will be able to actually show quantifiable temperature increases or decreases when used with a living being.

For clinical applications involving human patients with a standard starting temperature of 37 °C it might be necessary to personalize the calibration process. There is also the possibility, and likely the need, to combine our experiments with other image based temperature measurements, like quantitative ultrasound (QUS) that can be used to quantify tissue microstructure giving rise to scattered ultrasound^15^.

We are very excited about the possibility to not only obtain and identify tissue characteristics, detect events, but to also potentially receive tissue temperature changes by just analyzing the vibroacoustic signals generated by that motion.

We invite other research teams to work on advancing this promising approach for measuring temperature and make the annotated audio data sets available for download and unrestricted use (see link below).

## Data Availability

We happily invite scientists to improve this novel and promising technology approach.The audio data files are available for download and unrestricted use (cite the authors and institution) on HuggingFace (https://huggingface.co/datasets/VibroNav/VibroTemp092024). There are 359 files each for the audio and annotations, as well as 4.393 spectrogram slices (100ms width over the entire frequency spectrum) ordered by temperature range (20 to 55 in steps of 5). For issues related to the data files you may contact W.S.

## Declarations

### Use of LLM

Large Language Models like ChatGPT were not used for writing and editing of this manuscript.
